# Identification and characterization of two new 5-keto-4-deoxy-D-Glucarate Dehydratases/Decarboxylases

**DOI:** 10.1186/s12896-016-0308-3

**Published:** 2016-11-17

**Authors:** André Pick, Barbara Beer, Risa Hemmi, Rena Momma, Jochen Schmid, Kenji Miyamoto, Volker Sieber

**Affiliations:** 1 Technical University of Munich, Straubing Center of Science, Chair of Chemistry of Biogenic Resources, Schulgasse 16, 94315 Straubing, Germany; 2 Department of Biosciences and Informatics, Keio University, 3-14-1 Hiyoshi, 2238522 Yokohama, Japan

**Keywords:** Keto-deoxy-D-Glucarate, *Acinetobacter baylyi*, *Comamonas testosteroni*, *Polaromonas naphthalenivorans*, Dehydratase

## Abstract

**Background:**

Hexuronic acids such as D-galacturonic acid and D-glucuronic acid can be utilized via different pathways within the metabolism of microorganisms. One representative, the oxidative pathway, generates α-keto-glutarate as the direct link entering towards the citric acid cycle. The penultimate enzyme, keto-deoxy glucarate dehydratase/decarboxylase, catalyses the dehydration and decarboxylation of keto-deoxy glucarate to α-keto-glutarate semialdehyde. This enzymatic reaction can be tracked continuously by applying a pH-shift assay.

**Results:**

Two new keto-deoxy glucarate dehydratases/decarboxylases (EC 4.2.1.41) from *Comamonas testosteroni* KF-1 and *Polaromonas naphthalenivorans* CJ2 were identified and expressed in an active form using *Escherichia coli* ArcticExpress(DE3). Subsequent characterization concerning *K*_m_, *k*_cat_ and thermal stability was conducted in comparison with the known keto-deoxy glucarate dehydratase/decarboxylase from *Acinetobacter baylyi* ADP1. The kinetic constants determined for *A. baylyi* were *K*_m_ 1.0 mM, *k*_cat_ 4.5 s^−1^, for *C. testosteroni K*_m_ 1.1 mM, *k*_cat_ 3.1 s^−1^, and for *P. naphthalenivorans K*_m_ 1.1 mM, *k*_cat_ 1.7 s^−1^. The two new enzymes had a slightly lower catalytic activity (increased *K*_m_ and a decreased *k*_cat_) but showed a higher thermal stability than that of *A. baylyi*. The developed pH-shift assay, using potassium phosphate and bromothymol blue as the pH indicator, enables a direct measurement. The use of crude extracts did not interfere with the assay and was tested for wild-type landscapes for all three enzymes.

**Conclusions:**

By establishing a pH-shift assay, an easy measurement method for keto-deoxy glucarate dehydratase/decarboxylase could be developed. It can be used for measurements of the purified enzymes or using crude extracts. Therefore, it is especially suitable as the method of choice within an engineering approach for further optimization of these enzymes.

**Electronic supplementary material:**

The online version of this article (doi:10.1186/s12896-016-0308-3) contains supplementary material, which is available to authorized users.

## Background

Renewable biogenic resources such as lignocellulosic hydrolysates, often referred to as second-generation feedstock, represent an increasingly important raw material for chemicals production. Complete exploitation of these substrates is still a challenging task due to their heterogeneous composition. Besides various hexoses and pentoses, which constitute the main fraction of the hydrolysates, sugar derivatives such as sugar acids accumulate. The latter include hexuronic acids such as D-galacturonic acid and D-glucuronic acid, which are mainly present when pectin-rich waste streams or plant xylans are utilized [1, 2]. Both acids are abundantly available in agricultural waste or forestry residues. In particular, plant pathogenic bacteria such as *Pseudomonas syringae*, *Agrobacterium tumefaciens* or *Erwinia carotovora* as well as *Escherichia coli* or *Thermotoga maritima* possess metabolic pathways for hexuronic acid utilization [3–7]. Up to now, three pathways have been identified for the utilization of hexuronic acids via isomerization, reduction or oxidation [8].

**Fig. 1:**
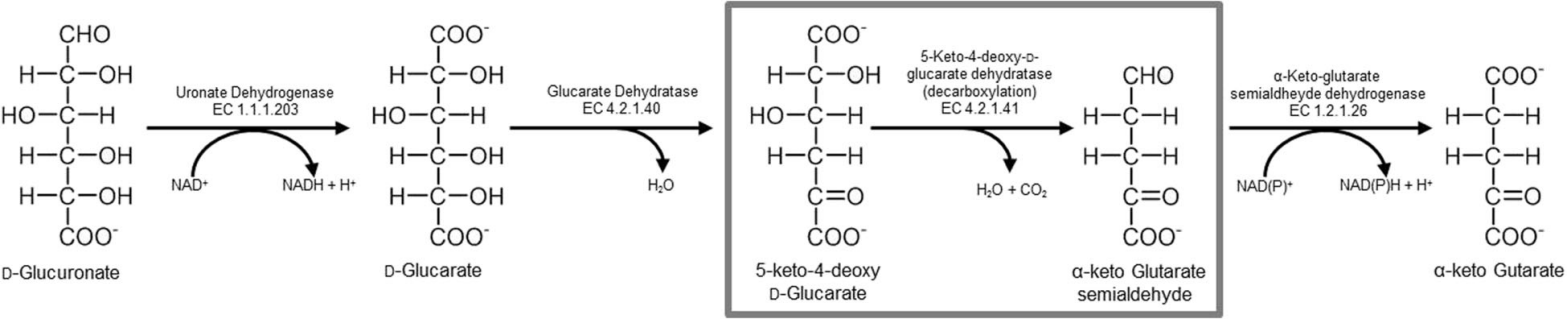
Oxidative Pathway. Schematic representation of the oxidative pathway for conversion of uronic acids using D-glucuronate as starting substrate

The oxidative pathway comprises four enzymatic steps (Fig. 1), generating α-keto-glutarate as the direct link entering the citric acid cycle [8]. The first oxidative step is catalysed by uronate dehydrogenase, which produces an aldaric acid lactone that hydrolyses spontaneously [9, 10]. Several uronate dehydrogenases of different origins have been described [11–14].

The subsequent two steps are catalysed by the enzymes glucarate dehydratase and keto-deoxy glucarate dehydratase/decarboxylase (KdgD). Both enzymes are responsible for the defunctionalisation of glucarate. First, glucarate dehydratase removes water, leading to keto-deoxy glucarate, which is the substrate for KdgD; this in turn catalyses the dehydration and decarboxylation into α-keto-glutarate semialdehyde [15]. In the final step, α-keto-glutarate semialdehyde dehydrogenase oxidizes the semialdehyde to α-keto-glutarate [16]. The glucarate dehydratase belongs to the mechanistically diverse enolase superfamily, which is known to catalyse at least 14 different reactions [17]. Within this superfamily, glucarate dehydratase is assigned to the mandelate racemase subgroup [18]. The reaction mechanism and protein structure of several members have been studied in detail [19, 20]. The bifunctional enzyme KdgD belongs to the class I aldolase family and is further sub-grouped into the *N*-acetylneuraminate lyase superfamily [21]. Only little attention has been devoted towards this enzyme even though it catalyses a very interesting reaction. Just recently, the crystal structure for KdgD from *A. tumefaciens* was solved [22] in parallel with investigations to gain a deeper understanding of the catalytic mechanism, which led to the identification of catalytically relevant amino acids [23].

For thorough characterization, easy monitoring of the enzymatic reaction is one of the main challenges. Neither the substrate nor the product can be detected photometrically; moreover, no cofactor is involved in the catalytic reaction. Therefore, all studies performed up to now have used a coupled enzyme assay with α-keto-glutarate semialdehyde dehydrogenase, following the formation of NAD(P)H at 340 nm [15]. However, the reaction catalysed by KdgD is well suited to establish a direct method for measuring enzymatic activity. The release of CO_2_ from a carboxylate leads to the consumption of protons and an increase in pH, which in theory can be monitored by a pH indicator and no additional enzyme is necessary to detect the reaction. Colorimetric assays based on a pH indicator system have been successfully used to monitor several enzymatic reactions, e.g. hydrolysis of esters, transfer of sugars, phosphate or nucleotides, as well as decarboxylation of amino acids [24–30].

Here, we report the identification and characterization of two novel KdgDs from *Comamonas testosteroni* KF-1 (Ct) and *Polaromonas naphthalenivorans* CJ2 (Pn). For better evaluation and validation, an already known KdgD from *Acinetobacter baylyi* ADP1 (Ab) was used as the reference. A first characterization and comparison was done by developing an easy and direct measurement method based on a pH indicator system using bromothymol blue (BTB) as the indicator and potassium phosphate as the buffer. The assay could be easily adopted to allow measurements in crude cell extracts and therefore will be very useful for screening approaches.

## Methods

### Reagents

D-Saccharic acid potassium salt (glucarate), magnesium chloride heptahydrate and BTB sodium salt were purchased from Sigma Aldrich (Seelze, Germany). Restriction enzyme BsaI, alkaline phosphatase, Phusion™ polymerase, T4 ligase and T4 polynucleotide kinase were purchased from New England Biolabs (Frankfurt, Germany). Taq polymerase was obtained from Rapidozym (Berlin, Germany). Oligonucleotides were synthesized by Thermo Fisher Scientific (Waltham, MA, USA). DNaseI was obtained from Applichem (Darmstadt, Germany). All other chemicals were purchased from Carl Roth (Karlsruhe, Germany) or Merck (Darmstadt, Germany) and were used without further purification. All columns used for protein purification were from GE Healthcare (Munich, Germany).

### Sequence selection and comparison

The publicly available protein sequence of the 5-keto-4-D-deoxyglucarate dehydratase/decarboxylase of *A. baylyi* ADP1 was used as the query sequence for BLAST analysis (blastp) for the identification of potential candidates [31]. Candidate proteins belonging to another species with a maximum identity of 70 % were chosen. In the next step, to verify the possible occurrence of the oxidative pathway for D-glucuronate and D-glucarate conversion, the occurrence of the upstream enzyme D-glucarate dehydratase was confirmed by screening the genome sequence of *P. naphthalenivorans* CJ2 (NC_008781.1) and *C. testosteroni* KF-1 (AAUJ02000001.1). Identification of both enzymes was the final criterion for selection.

Four protein sequences encoding for KdgD were aligned using the web-based program T-COFFEE. 5-keto-4-D-deoxyglucarate dehydratase/decarboxylase of *A. baylyi* ADP1, the enzyme from *A. tumefaciens* C58, whose structure was recently determined, were chosen as references [23]. Based on the BLAST results, the two enzyme candidates from *P. naphthalenivorans* CJ2 (WP_011800997.1) and *C. testosteroni* KF-1 (WP_003059546.1) were chosen.

### Strains and plasmids

The following strains were used in this study: *E. coli* XL1-Blue (Stratagene), *E. coli* BL21(DE3) (Novagen) and *E. coli* ArcticExpress(DE3) (Agilent Technologies). The DNA sequences for the corresponding genes of keto-deoxy-D-glucarate dehydratase/decarboxylase (kdgD) from *A. baylyi* (ADP1) (protein sequence GenBank™ WP_004930673.1), which is identical to *A. baylyi* DSM 14961 (protein sequence GenBank™ ENV53020.1), from *C. testosteroni* KF-1 (protein sequence GenBank™ WP_003059546.1) and from *P. naphthalenivorans* (protein sequence GenBank™ WP_011800997.1) were synthesized with optimized codon usage for expression in *E. coli* (Additional file 1: Figures S1-S3) (Life Technologies, Regensburg, Germany). The following primers were used for amplification: F-kdgD-A.b.- CAGCAA**GGTCTCA**CATATGGATGCCCTGGAACTG, R-kdgD-A.b.-CTGCGG-ACCCAGGGTTG, F-kdgD-C.t.-CAGCAA**GGTCTCA**CATATGACACCGCAGG-ATCTGAAAG, R-kdgD-C.t.-xCTGCGGACCCAGTTTATCAATC, F-kdgD-P.n.- CAGCAA**GGTCTCA**CATATGAATCCGCAGGATCTGAAAAC, R-kdgD-P.n.- CTGCGGACCCAGGCTTTTAATC. The restriction enzyme recognition site for BsaI is underlined and the start codon is marked in bold. The reverse primers were phosphorylated using T4 polynucleotide kinase according to the supplier’s manual. PCR products were digested with BsaI and cloned into pCBR, pCBRHisN and pCBRHisC, which are derivatives of pET28a (Novagen). The cloning strategy of all pET28 derivatives is described by Guterl et al. [32]. Ligation of the PCR products and the following transformation led to the plasmids pCBR-kdgD-A.b., pCBR-kdgD-C.t., pCBR-kdgD-P.n., pCBRHisN-kdgD-A.b., pCBRHisN-kdgD-C.t., pCBRHisN-kdgD-P.n., pCBRHisC-kdgD-A.b., pCBRHisC-kdgD-C.t., and pCBRHisC-kdgD-P.n. Multiplication of the plasmids was performed by *E. coli* XL1 Blue (Stratagene) in Luria–Bertani medium containing 30 μg/ml kanamycin. *E. coli* BL21(DE3) or *E. coli* ArcticExpress(DE3) were used for expression.

### Overexpression and FPLC purification

Protein expression was performed with two different *E. coli* expression strains depending on the target enzyme. *E. coli* BL21(DE3) [pET28a-NH-kdgD-A.b.] was cultivated with a slightly modified protocol described by Aghaie et al. [15]. The preculture was incubated in 4 ml of Terrific broth medium supplemented with 1 M sorbitol and 5 mM betaine with 100 μg/ml kanamycin at 37 °C overnight on a rotary shaker (180 rpm). The expression culture consisted of the same media and was inoculated with a 1:100 dilution of the preculture. Incubation was performed at 37 °C until an OD_600_ of 2 was reached. Protein expression was induced with the addition of IPTG to a final concentration of 0.5 mM followed by incubation for 21 h at 16 °C. For *E. coli* ArcticExpress(DE3) [pET28a-NH-kdgD-C.t. or pET28a-CH-kdgD-P.n.] the preculture was cultivated in Luria–Bertani media with 100 μg/ml kanamycin and 15 μg/ml gentamycin over night at 37 °C. The expression culture consisted of autoinduction media with both antibiotics and was inoculated with a 1:100 dilution of the preculture [33]. Incubation was performed for 3 h at 37 °C followed by the second step at 12 °C for 45 h. Afterwards, cells were harvested by centrifugation and washed one time with 50 mM sodium phosphate buffer (pH 8.0) and frozen at −20 °C or resuspended in a binding buffer (50 mM potassium phosphate, pH 8.0, 20 mM imidazol, 500 mM NaCl and 10 % glycerol). Crude extracts were prepared using a Basic-Z cell disrupter (IUL Constant Systems) and the subsequent addition of MgCl_2_ to a final concentration of 2.5 mM in combination with DNaseI (10 μg/ml), followed by an incubation for 20 min at room temperature for successful DNA degradation. The insoluble fraction of the lysate was removed by centrifugation at 20,000 rpm for 20 min at 4 °C. The supernatant was filtered through a 0.45 μm syringe filter and applied to an IMAC affinity resin column, 5 ml HisTrapTM FF, equilibrated with the binding buffer using the ÄKTA purifier system. The enzyme was washed with 20 ml of binding buffer and eluted with 50 mM potassium phosphate buffer (pH 8.0, 500 mM imidazol, 500 mM NaCl and 10 % glycerol). Aliquots of each eluted fraction were subjected to 12 % SDS-PAGE. The fractions containing the eluted protein were pooled and the protein was desalted using a HiPrepTM 26/10 desalting column, which was preliminary equilibrated with 50 mM Tris-HCl (pH 7.5) or 5 mM sodium phosphate buffer (pH 7.0). Protein concentrations were determined using a Bradford assay (Roti®-nanoquant, Carl Roth).

### Enzyme expression in 96-deep well scale

For all three enzymes, an expression in the 96-deep well scale was performed. Therefore, electrocompetent *E. coli* ArcticExpress(DE3) cells were transformed with the corresponding plasmid. Single clones were picked using the CP7200 Colony Picker (Norgren Systems) and transferred to 96-deep well plates filled with 1.2 ml autoinduction media [33] by a MicroFlo Select dispenser (Bio-Tek Instruments). After incubation (36 h, 37 °C at 1,000 rpm), further processing was done manually. First, 100 μl of cell culture was transferred into a 96-well plate (U-shaped bottom) and harvested by centrifugation (4,570 rpm, 10 min at RT) while the supernatant was discarded. The frozen pellets (1 h at −20 °C) were thawed at room temperature for one hour to improve cell lysis. Lysis was continued by the addition of 30 μl lysis buffer (3 h, 1,000 rpm, 37 °C) containing 2 mM KP_i_, pH 7.0, 2 mM MgCl_2_, 10 μg/ml DNaseI, 100 μg/ml lysozyme. Next, 120 μl buffer (2 mM KP_i_, pH 7.0) was added followed by centrifugation (3,000 rpm, 15 min at RT). For the photometric measurement, 20 μl of the crude extract was transferred to a 96-well plate (F-shaped bottom) and the reaction was started by adding 180 μl master mix to give a final volume of 200 μl (2.5 mM KP_i_, pH 7.0, 2 mM MgCl_2_, 25 μg/ml BTB and 5 mM keto-deoxy-D-glucarate). The measurements were carried out for 60 min at 2-min intervals. Depending on the enzyme, different time windows were used for the activity calculation.

### Substrate preparation

5-keto-4-deoxy-D-glucarate is not commercially available. Therefore, it was prepared using an enzymatic conversion of D-glucarate. For that, a 250–500 mM solution of D-glucarate containing 2 mM MgCl_2_ was prepared. Potassium glucarate is not completely soluble at this concentration, and the pH value was around 4.5. The pH was shifted to 8.0 by adding potassium hydroxide. A sample was taken as zero-point control and the reaction was started by the addition of D-glucarate dehydratase (Beer et al., manuscript in preparation). Using HPLC, a sample was analysed at regular intervals. After full conversion, the reaction was stopped by removing the enzyme by filtration (spin filters, 10 kDa MWCO, modified PES; VWR, Darmstadt, Germany). In the last step, the pH was adjusted to 7.0 using HCl.

### HPLC analysis

D-glucarate, 5-keto-4-D-deoxyglucarate and α-ketoglutarate semialdehyde were separated by HPLC, using an Ultimate-3000 HPLC system (Dionex, Idstein, Germany), equipped with an autosampler (WPS 3000TRS), a column compartment (TCC3000RS) and a diode array detector (DAD 3000RS). The column Metrosep A Supp10–250/40 column (250 mm, particle size 4.6 mm; Metrohm, Filderstadt, Germany) at 65 °C was used for separation by isocratic elution with 30 mM ammonium bicarbonate (pH 10.4) as the mobile phase at 0.2 mL min^−1^. Samples were diluted in water, filtered (10 kDa MWCO, modified PES; VWR, Darmstadt, Germany) and 10 μL of the samples was applied on the column. Data was analysed with Dionex Chromelion software.

### Determination of Δε_617_ for BTB and Q factor calculation

The extinction coefficient difference Δε_617_ was determined experimentally. The protonated form of BTB (0–100 μg/mL) was measured in different potassium phosphate buffer concentrations (2.5–10 mM) at pH 5.5. In addition, the deprotonated form was measured at pH 8.0 under identical conditions. For both measurements, the concentration multiplied by the pathlength ((mol/L)⨯(cm)) was plotted against the absorbance and the slope was determined. Δε_617_ was calculated by subtracting the value of the protonated BTB from the deprotonated BTB.

After determination of Δε_617_, the buffer factor Q factor, a constant relating absorbance change and reaction rate for a given buffer/indicator system [30, 34, 35], was calculated for different buffer and indicator concentrations by using equation (1). C_B_ and C_In_ are the total molar concentrations of the buffer and the indicator, respectively, and l represents the path length.

1 $$ Q=\frac{C_B/{C}_{In}}{\varDelta_{\varepsilon_{617}\times l}} $$

### Colorimetric assay

For direct detection of KdgD activity, a colorimetric assay in a 96-well microplate format was developed in a Multiskan® spectrum spectrophotometer (Thermo Fisher Scientific). The total reaction volume was 200 μl and consisted of 2.5 mM potassium phosphate buffer (pH 7.0), 2 mM MgCl_2_, 25 μg/ml BTB and the substrate at 37 °C. Every measurement was conducted at least three times. Addition of the enzyme solution initiated the measurement. Enzyme concentration for each KdgD varied and corresponded to a suitable signal over time. One unit of enzyme activity was defined as the amount of protein that converts 1 μmol of substrate/min at 37 °C. Calculation of the enzyme velocity was performed using equation (2), where d*A*/d*t* is the rate of absorbance change, V_R_ and V_E_ represent the reaction volume and the enzyme volume, c_E_ is the enzyme concentration and D is a measure of the dilution factor for the enzyme solution. The enzyme concentration allowed use of an 8–10 min time window for a linear slope. Substrate conversion was always below 10 % for each concentration during the kinetic measurements.

2 $$ U/ mg=\frac{\frac{\mathrm{d}A}{\mathrm{d}t}\times Q\times {V}_R\times D}{V_E\times {C}_E} $$

### Enzyme characterization

Each enzyme was investigated concerning *K*_m_ and *k*_cat_. The substrate concentration for the kinetic measurements was in the range 0.05–20 mM. The other conditions remained the same as was described in the previous section for the colorimetric assay. Calculation of the Michaelis-Menten kinetic parameters was done by fitting the data to the Michaelis-Menten equation (3)

3 $$ \boldsymbol{v}=\frac{{\mathbf{v}}_{\boldsymbol{m}\boldsymbol{ax}}\times \left[\boldsymbol{s}\right]}{{\boldsymbol{K}}_{\boldsymbol{m}}\times \left[\boldsymbol{s}\right]} $$

using Sigma–Plot 11.0 (Systat Software). The Michaelis-Menten equation consists of the following terms: *v* is the reaction rate (μmol/min/mg), *V*_max_ is the maximum reaction rate (μmol/min/mg), *[S]* is the varying substrate concentration (mM), and *K*_m_ is the Michaelis-Menten constant (mM).

The enzyme stability of the variants was investigated for storage at 8 °C (refrigerator) and in the context of cryo-conservation and reuse. Enzyme stock solutions in 50 mM Tris-HCl (pH 7.5) were stored without additional cryo-protectants, such as glycerol, at −20 °C.

Enzyme stability was investigated using two different temperatures: 37 °C and 65 °C. Therefore, aliquots of each enzyme for each measuring point with a volume of 100 μl were incubated in a water bath. The enzymes were incubated using a 5 mM KPi buffer (pH 7.0) at a concentration of 0.13 mg/ml. Therefore, the storage buffer 50 mM Tris-HCl (pH 7.5) was exchanged with the ÄKTA purifier system using a HiPrepTM 26/10 desalting column. In case of KdgDAb, an additional buffer system of 10 mM NH_4_HCO_3_ (pH 7.9) was used. For the measurements, 5 mM substrate was used. The half-life for each enzyme at the incubation temperature of 65 °C was calculated according to Rogers and Bommarius [36].

## Results and discussion

### Selection of KdgDs

**Fig. 2:**
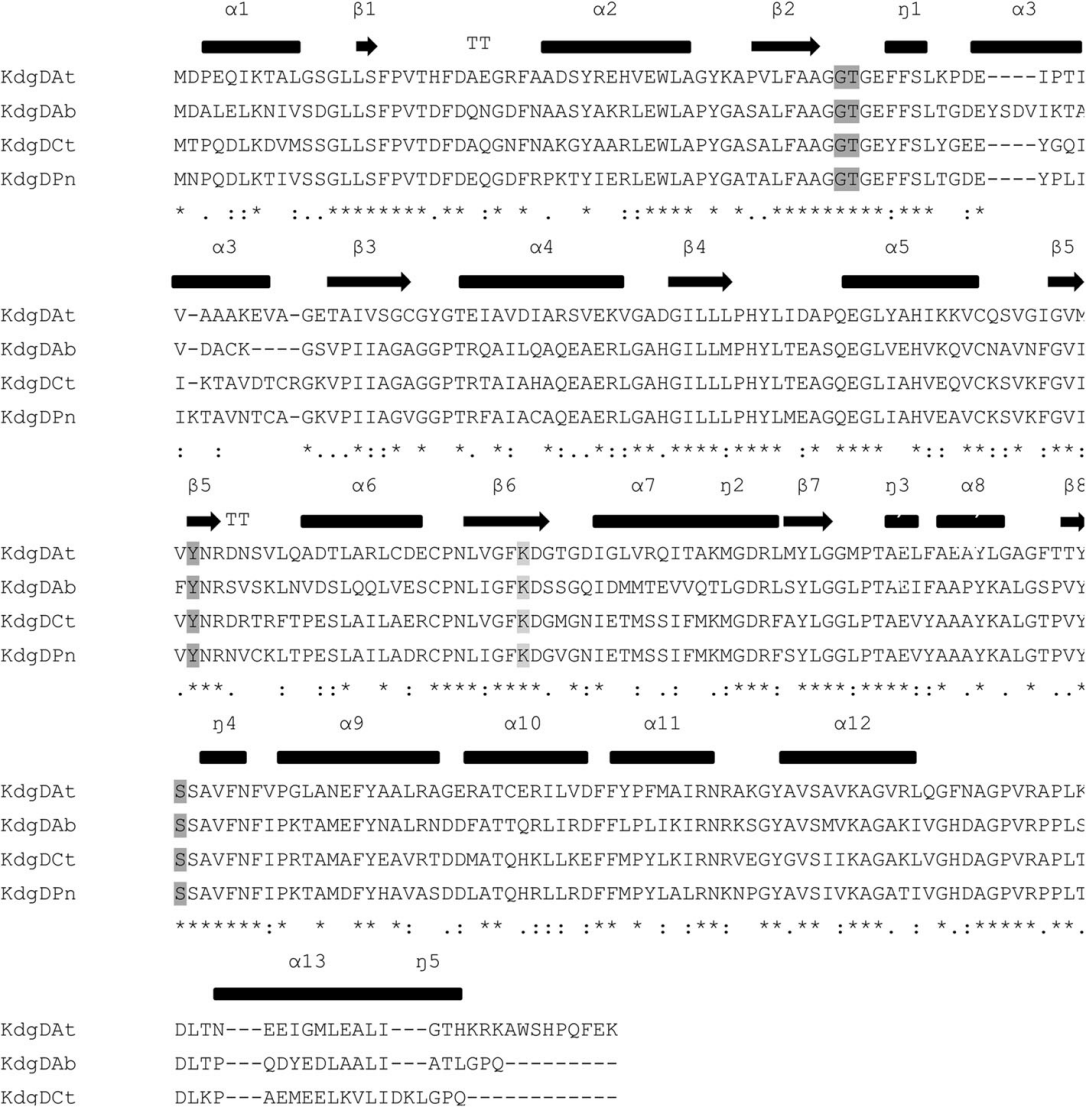
Sequence alignment. Multiple sequence alignment of known 5-keto-4-deoxy-D-glucarate dehydratases/decarboxylases using clustal omega [46]. The secondary structures are shown above with thick bars representing α-helices and arrows representing β-sheets. Coloured residues represent conserved residues of the active centre involved in the specific substrate recognition. The lysine residue (light grey) forms the intermediate Schiff base with the substrate. The sequence identity for all three dehydratases/decarboxylases obtained by referring to KdgDAt as the standard and performing pairwise alignment with EMBOSS Needle [40] are as follows: KdgDAb = 47 %, KdgDCt = 49 %, KdgDPn = 52 %

**Fig. 3:**
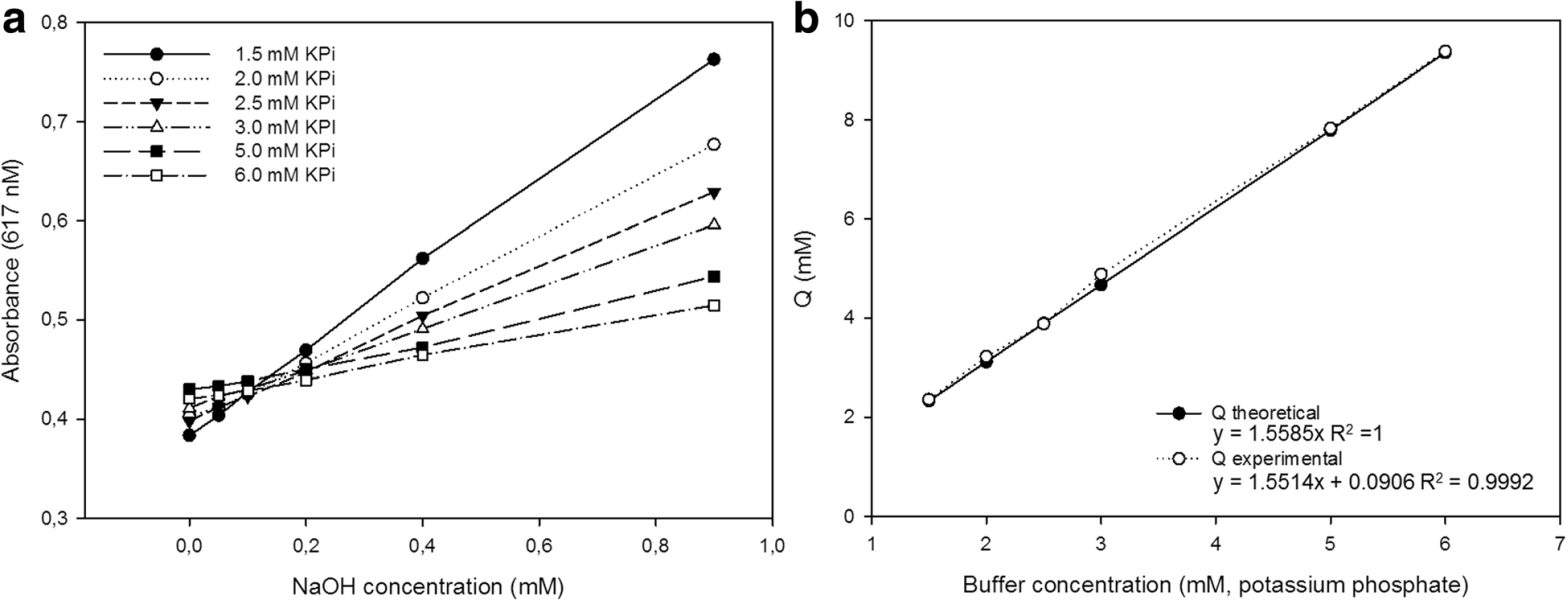
pH-dependent assay development. **a** Determination of Q factor with respect to different buffer concentrations. Absorbance *E*_617nm_ was linear for all tested buffer concentrations within the given sodium hydroxide concentration. **b** Comparison of theoretical (Q_thr_) and experimental (Q_exp_) Q factors to determine accuracy of the assay

Until now, only a few KdgD enzymes are described in the literature [37–39]. Recently, the complete oxidative pathway for *A. baylyi* ADP1 was elucidated and the enzymes involved were recombinantly expressed in *E. coli* [15]. Therefore, the selection of the novel KdgD candidates was performed by a BLAST analysis based on the amino acid sequence of the known enzyme derived from *A. baylyi* ADP1 identified by Aghaie et al. [15]. Two candidates were chosen, showing less than or equal to 70 % identity toward *A. baylyi* ADP1 based on a pairwise alignment performed by EMBOSS Needle [40], *P. naphthalenivorans* CJ2 (69.6 %) and *C. testosteroni* KF-1 (67.7 %) (Fig. 2). With an eightfold (βα) barrel structure, these enzymes share a ubiquitously found motif that is capable of catalysing many different reactions [41]. As a member of the *N*-acetylneuraminate lyase superfamily, KdgD exhibits a conserved lysine residue (Fig. 2, light grey) that forms the Schiff base essential for the enzymatic reaction at the end of the sixth β-sheet. Furthermore, a tyrosine residue located at the end of the fifth β-sheet (Fig. 3, dark grey) is also conserved and catalyses the deprotonation of the β-carbon after the Schiff base had been formed. In the next step, the hydroxyl group at the fourth carbon atom is protonated and subsequently released as a water molecule; this is mediated by a serine positioned at the C-terminus of the eighth β-sheet. A glycine and threonine (Fig. 3, dark grey) at the end of the second β-sheet coordinate the C_6_-carboxylate group. The conserved residues described by Taberman et al. can be found in all investigated enzymes [23].

### Heterologous expression

The codon-optimized kdgD genes of *A. baylyi*, *C. testosteroni* and *P. naphthalenivorans* were heterologously expressed in different *E. coli* expression strains. Untagged versions as well as His-tagged versions (oH = without His-Tag, CH = C-terminal His-Tag and NH = N-terminal His-Tag) were constructed for all genes of interest. Using *E. coli* BL21(DE3) with autoinduction media resulted in inclusion bodies for all three enzymes in every His-tag as well as without His-tag version. Therefore, the expression using slightly modified conditions that was described by Aghaie et al. was used [15]. Terrific broth medium containing 1 M sorbitol and 5 mM betaine was used for the expression starting at 37 °C until A600 reached ≥1.5. After the addition of IPTG to a final concentration of 1 mM, cultivation was continued at 16 °C for 20 h. Under these conditions, a soluble expression of the NHKdgDAb (hereafter, referred as KdgDAb), oHKdgDAb and oHKdgDCt was possible. Using an ArcticExpress(DE3) *E. coli* expression strain in combination with autoinduction media enabled the expression of NHKdgDCt (KdgDCt) and CHKdgDPn (KdgDPn) in a soluble form. The distribution between the insoluble and the soluble proteins varied from almost complete solubility of NHKdgDAb to an 80:20 ratio for NHKdgDCt and CHKdgDPn (data not shown). Eluted proteins appeared as a single band on SDS polyacrylamide gels and no further bands indicating co-elution of chaperonins of *E. coli* ArcticExpress(DE3) expression were detected. This is notwithstanding the publication of several reports that mention co-elution as a problem for successful enzyme purification [42, 43]. Enzyme activities of the different KdgDs were stable after storage at 8 °C in desalting buffer (50 mM Tris-HCl, pH 7.5) for at least 14 days. Independent stable long-term storage, preserving the catalytic activity of the enzymes, (four month) at −20 °C and −80 °C was realized without cryo-protectants.

### Assay validation

A reliable and sensitive pH-shift assay requires the pKa of the indicator and the buffer to be very similar to allow a direct correlation between the colour change and the changing concentration of hydrogen ions within the assay solution. The pKa of the phosphate buffer is 7.2 and that of the BTB lies between 7.1 and 7.3, resulting in a suitable combination.

The absorbance spectra of protonated and deprotonated BTB were determined and the maximum difference in extinction coefficient was observed at 617 nm, which compares well with the wavelength reported in the literature [44]. The large difference in the extinction coefficient between protonated and deprotonated BTB (Δε_617_ 28101 M^−1^ cm^−1^) results in a low Q value (Eq. 1), and this in turn ensures a high sensitivity (dA/dt) of the assay. For a final validation, the buffer factor (Q) was calculated and determined experimentally to guarantee the reliability of the assay. Q was experimentally determined (Q_exp_) by testing several buffer concentrations and titrations of sodium hydroxide in the range of 0–1 mM (Fig. 3a). The reciprocal of the slopes directly correlates to Q, and the theoretical Q (Q_thr_) value for each buffer concentration was calculated using equation (1). Buffer concentration was plotted against Q_thr_ and Q_exp_ to check the extent of the correlation (Fig. 3b). Although a concentration of 1.5 mM resulted in almost the same Q_thr_ and Q_exp_, a higher buffer concentration of 2.5 mM was chosen. The reasons for this are an increased robustness of the assay and better pH stability in the initial phase of the measurement. The Q value at this buffer concentration was still sufficiently low to guarantee high sensitivity.

### Enzyme characterization

**Fig. 4:**
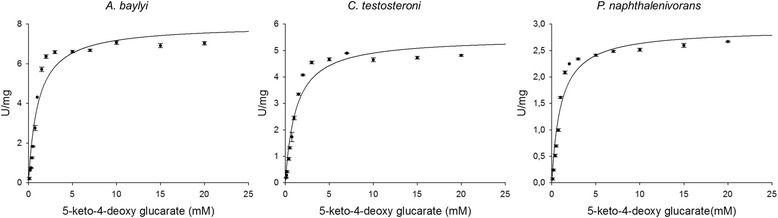
Graphical representations of kinetic measurements. Conditions: 37 °C, 2.5 mM KP_i_ buffer (pH 7.0), 2 mM MgCl_2_ and 25 μg/ml BTB

The purified enzymes were used to determine their kinetic parameters *k*_cat,_ *K*_m_ and v_max_ for the substrate keto-deoxy glucarate (Table 1). The enzyme KdgDAb characterized by Aghaie et al. using a coupled enzyme method [15] was used to allow comparison of the same parameters obtained here using the pH-shift assay and to compare with the activity of KdgDCt and KdgDPn. The *K*_m_ for keto-deoxy glucarate was almost similar for all three enzymes (1.0–1.1 mM). The graphs for determination of the Michaelis–Menten constants are shown in Fig. 4. There were differences in *k*_cat,_ and v_max_ among the three enzymes. KdgDAb showed the highest *k*_cat_ with 4.5 s^−1^ followed by KdgDCt (3.1 s^−^1) and KdgDPn (1.7 s^−1^). The kinetic parameters for KdgDAb differ slightly from those reported by Aghaie et al., especially the higher *K*_m_, but also *k*_cat_. The latter appear to be similar, however Aghaie et al. and Taberman et al. measured the enzymatic activity at a lower temperature (22 °C) compared to the pH-shift assay (37 °C) indication a 2-fold lower *k*_cat_ value obtained within this study [15, 23]. This difference might be explained by the differing buffer conditions during the kinetic parameter measurement. The different buffer system as well as the reduced buffer concentration without any stabilizers (glycerol, DTT or NaCl) in combination with a slight decrease in the pH value might be responsible for this observation. Taberman et al. described the KdgD of *A. tumefaciens* and identified the pH optimum to be in the range 7.5–8.0 [23]. For this measurement, a phosphate buffer (pH 7.5) with additional NaCl was used and a *K*_m_ of 0.5 mM was reported. The enzyme showed only 65 % activity at pH 7.0 compared with the activity at pH 8.0 (12 U/mg) using a phosphate buffer. Application of the pH-shift assay system using BTB/phosphate buffer at a higher pH is not suitable.

**Table 1:** Kinetic parameters determined for KdgDAb, KdgDCt and KdgDPn at 37 °C using the pH-shift assay with 2.5 mM KP_i_ buffer (pH 7.0)

Enzyme	*K*_m_ [mM]	*k*_cat_ [1/s]	*k*_cat_/*K*_m_ [1/mM s]	v_max_ [U/mg]
KdgDAb	1.0 ± 0.1	4.5 ± 0.02	4.5	8.0 ± 0.27
KdgDCt	1.1 ± 0.1	3.1 ± 0.02	2.8	5.3 ± 0.11
KdgDPn	1.1 ± 0.1	1.7 ± 0.01	1.5	3.0 ± 0.06
KdgDAt^a^	0.5	8.83 ± 0.16	17.0	
KdgDAb^b^	0.2	3.9 ± 0.04	19.2	

^a^ Assay conditions: 50 mM NaP_i_ pH 7.5, 100 mM NaCl, 4 mM NADP^+^, 22 °C [23]

^b^ Assay conditions: 50 mM Hepes/NaOH pH 7.5, 100 mM NaCl, 5 mM MgCl_2_, 4 mM NADP^+^, 10 % glycerol, 22 °C [15]

**Fig. 5:**
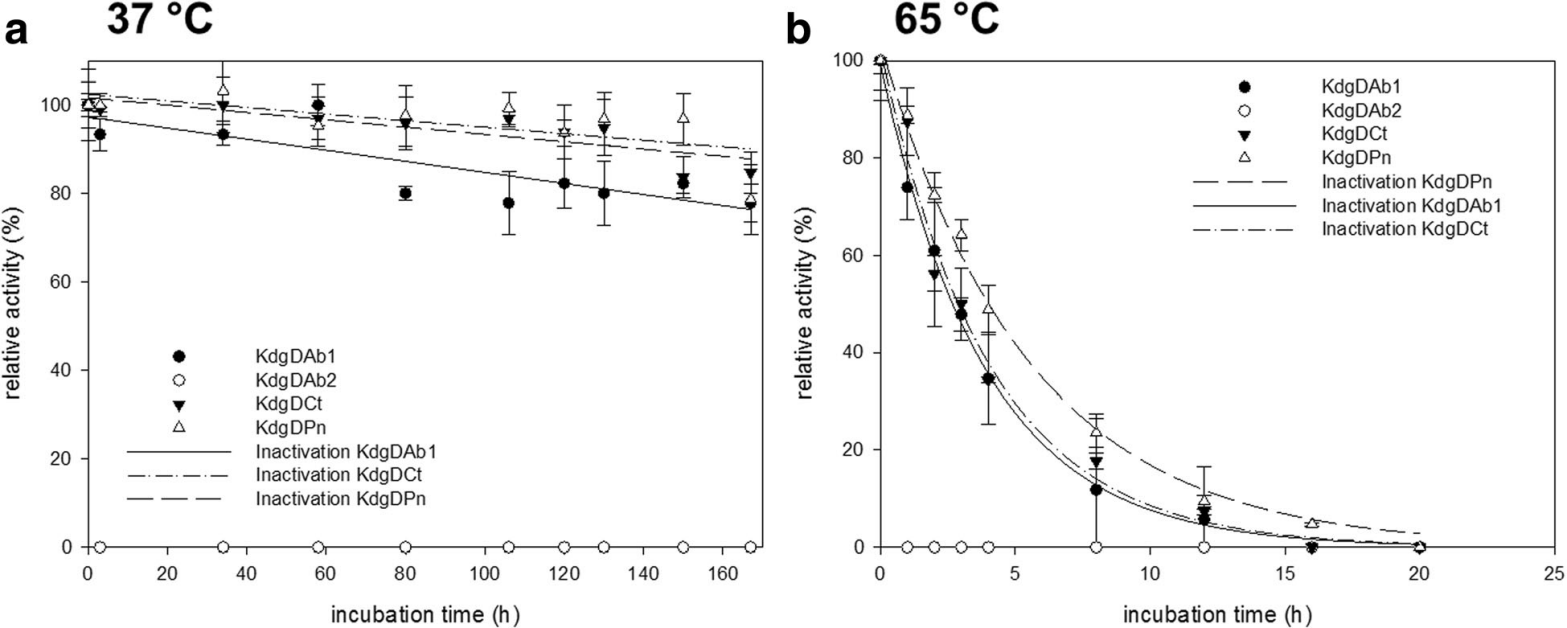
Temperature stability. **a** Incubation of KdgDAb1 (100 % = 6.22 U/mg), KdgDAb2 (100 % = 6.56 U/mg), KdgDCt (100 % = 5.5 U/mg) and KdgDPn (100 % = 3.7 U/mg) at 37 °C; **b** Incubation of KdgDAb1 (100 % = 8.22 U/mg), KdgDAb2 (100 % = 8.1 U/mg), KdgDCt (100 % = 8.4 U/mg) and KdgDPn (100 % = 5.1 U/mg) at 65 °C; assay: volume 200 μl, 2.5 mM KP_i_ (pH 7.0), 2 mM MgCl_2_, 25 μg/ml BTB and 5 mM 5-keto-4-D-deoxyglucarate; enzyme was incubated in 100 μl of 5 mM KP_i_ (pH 7.0); only KdgDAb1 differed, where additional 0.5 mM NH_4_HCO_3_ was used for enzyme stabilisation

In addition, the stability as a function of temperature was investigated. Activities of KdgDAb, KdgDCt and KdgDPn were determined at two different temperatures: 37 and 65 °C (Fig. 5a and b). KdgDCt and KdgDPn showed a similar behaviour at both temperatures. For both enzymes, activity was stable over 150 h at 37 °C, whereas after 4 h at 65 °C the initial activity decreased below 50 %. The calculated t_1/2_ was 2.79 h (KdgDCt) and 3.83 h (KdgDPn). KdgDAb (Fig. 5a, KdgDAb2) showed a different behaviour. A fast decrease in enzyme activity was observed already at 37 °C. It appeared that the KP_i_ buffer system had a negative influence on the stability of this enzyme. Changing the incubation conditions and adding an additional buffer at a low concentration, in this case, NH_4_HCO_3_ at a concentration of 0.5 mM, maintained stability of KdgDAb (Fig. 5a, KdgDAb1) at a similar level as seen for KdgDCt and KdgDPn. The calculated t_1/2_ at 65 °C for KdgDAb was 2.71 h and this is almost comparable to that of the other two enzymes. Additional, KdgDCt and KdgDPn were tested with NH_4_HCO_3_ at a concentration of 0.5 mM but no difference in stability or activity was detectable for both enzymes.

### High-throughput decarboxylase screening assay

After the verification of the pH-shift assay for the measurement of purified enzymes, its transferability to parallelization and HTS was tested by measuring Z-factor of the wild-type landscapes for all three KdgDs [45]. Again, *E. coli* ArcticExpress(DE3) was used as the expression strain for KdgDAb because of the positive results obtained for the expression of the other two KdgDs and in order to use the same standardized 96-well expression protocol for all three enzymes. Despite the fact that *E. coli* ArcticExpress(DE3) was developed to allow the expression of target proteins at a low temperature, the temperature was maintained constant at 37 °C. The reason for this was the inhomogeneous growth behaviour due to temperature switches during cultivation in 96-well scale observed for several different enzymes using autoinduction media. The cells were cultivated for 36 h to ensure that every expression culture reached their stationary phase. KdgDCt and KdgDPn showed a similar cell density at the end, whereas cell density of KdgDAb was lower compared with the other two strains (data not shown). The use of a commercial protein extraction reagent (B-PER, Thermo Fisher) did not improve cell lysis. Therefore, only DNaseI and lysozyme were used in the lysis step. After the lysis step, the lysate volume was increased to 150 μl to guarantee that no cell debris was transferred into the assay plate since the remaining cell debris showed a negative impact on the assay which resulted in a shift towards acidic pH conditions.

**Fig. 6:**
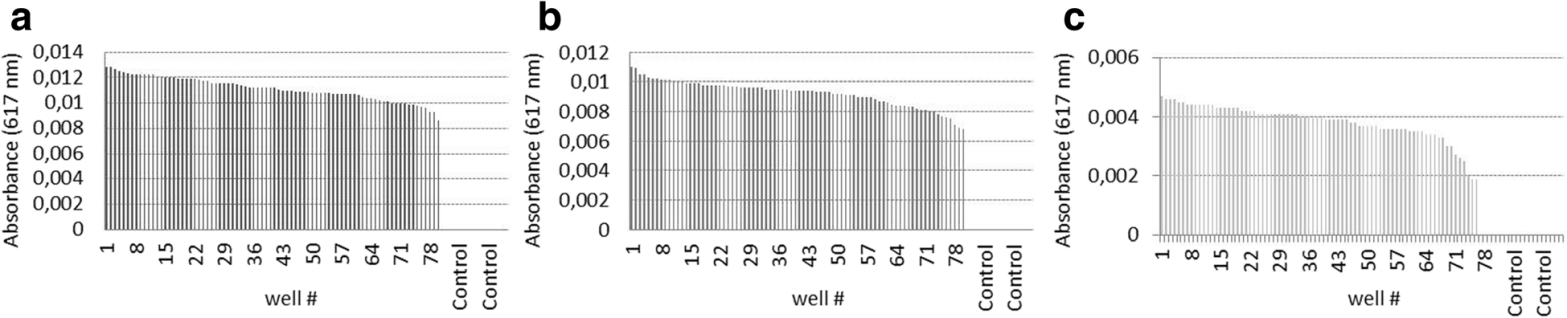
Wild-type landscapes for all three enzymes. Graphical representation of 96-well wild-type landscapes for all three enzymes. **a** KdgDAb (Z’ = 0.75), **b** KdgDCt (Z’ = 0.67), **c** KdgDPn (Z’ = 0.48); controls are empty vector and only media. Each 96-well plate contained 15 controls and 81 wells with the wild type enzyme. 100 μl of cell culture was centrifuged and the supernatant discarded. Lysis of the cells was done in 30 μl lysis buffer (2 mM KP_i_, pH 7.0, 2 mM MgCl_2_, 10 μg/ml DNaseI, 100 μg/ml lysozyme). Afterwards, 120 μl buffer (2 mM KP_i_, pH 7.0) was added and the lysed cells centrifuged. 20 μl of the crude extract was transferred into a new plate and 180 μl master mix was added to give a final volume of 200 μl (2.5 mM KP_i_, pH 7.0, 2 mM MgCl_2_, 25 μg/ml BTB and 5 mM keto-deoxy-d-glucarate)

Based on the pH-shift measurements, the Z’-factor was calculated for all three wild-type landscapes (Fig. 6). Z’ was 0.75 for KdgDAb, 0.67 for KdgDCt and 0.48 for KdgDPn. The linear slope was measured in a suitable range with an overall measurement time of 60 min. These findings are consistent with the activity of the enzymes discussed above. The Z’-values correspond well to the relative activities of the three enzymes. For screening purposes, a Z’-value of ca. 0.5 or above is desired. The developed method therefore was shown to be suitable for application in the directed evolution of all three dehydratase/decarboxylase enzymes.

## Conclusions

In conclusion, we identified two KdgD enzymes from *C. testosteroni* and *P. naphthalenivorans* and compared them with the already known enzyme from *A. baylyi* ADP1 concerning catalytic activity and stability. Therefore, we developed a pH-shift assay on the basis of BTB as the pH indicator and potassium phosphate as the buffer component. We were able to reduce the buffer concentration to 2.5 mM while maintaining reliability, reproducibility and sensitivity at a high level. KdgD enzymes from *C. testosteroni* (*K*_m_ 1.1 mM and *k*_cat_ 3.1 s^−^1) and *P. naphthalenivorans* (*K*_m_ 1.1 mM and *k*_cat_ 1.7 s^−1^) were characterized using this assay system. The calculated t_1/2_ was 2.79 h (KdgDCt) and 3.83 h (KdgDPn) at 65 °C. In addition, the assay system was successfully tested with crude extracts and a high reliability. Therefore, the 96-well based screening system enables further optimization of KdgD enzymes.

## Additional files

Additional file 1: Codon optimized nucleotide sequences for three kdgD genes coding for the keto-deoxy glucarate dehydratases/decarboxylases (EC 4.2.1.41) from *A. baylyi* ADP1 (**Figure S1**), *C. testosteroni* KF-1 (**Figure S2**), *P. naphthalenivorans* (**Figure S3**). (DOCX 34 kb)

## Abbreviations

BTB: Bromothymol blue
IMAC: Immobilized metal ion affinity chromatography
IPTG: Isopropyl-β-D-thiogalactopyranosid
KdgD: Keto-deoxy glucarate dehydratase/decarboxylase
MWCO: Molecular weight cut-off
