# Supplementary material for: Identification and characterization of two new 5-keto-4-deoxy-D-Glucarate Dehydratases/Decarboxylases
Source: BMC Biotechnol. 2016 Nov 17;16:80. doi: 10.1186/s12896-016-0308-3 (PMC5114784; doi:10.1186/s12896-016-0308-3)
Supplement: Additional file 1: — Codon optimized nucleotide sequences for three kdgD genes coding for the keto-deoxy glucarate dehydratases/decarboxylases (EC 4.2.1.41) from A. baylyi ADP1 (Figure S1), C. testosteroni KF-1 (Figure S2), P. naphthalenivorans (Figure S3). (DOCX 34 kb) [file 12896_2016_308_MOESM1_ESM.docx]

**Supplemental Information**

**Identification and characterization of two new 5-keto-4-deoxy-d-Glucarate Dehydratases/Decarboxylases André Pick^1^, Barbara Beer^1^, Risa Hemmi^2^, Rena Momma^2^, Jochen Schmid^1^, Kenji Miyamoto^2^, Volker Sieber^1^***

^1^ Technische Universität München, Wissenschaftszentrum Straubing, Lehrstuhl für Chemie Biogener Rohstoffe, Schulgasse 16, 94315 Straubing, Germany

^2^ Keio University, Department of Biosciences and Informatics, 3-14-1 Hiyoshi, 2238522, Yokohama, Japan

^*^ Correspondence: sieber@tum.de

**Figure S1** Codon optimized nucleotide sequence for the gene *kdg*D coding for the keto-deoxy glucarate dehydratases/decarboxylases (EC 4.2.1.41) from *A. baylyi* ADP1:

ATGGATGCCC TGGAACTGAA AAATATTGTG TCTGATGGTC TGCTGAGCTT TCCGGTTACC GATTTTGATC AGAATGGCGA TTTTAATGCA GCAAGCTATG CAAAACGTCT GGAATGGCTG GCACCGTATG GTGCAAGCGC ACTGTTTGCA GCCGGTGGCA CAGGTGAATT TTTTAGCCTG ACCGGTGATG AATATTCCGA TGTGATCAAA ACCGCAGTTG ATGCATGTAA AGGTAGCGTT CCGATTATTG CCGGTGCCGG TGGTCCGACC CGTCAGGCAA TTCTGCAGGC ACAGGAAGCA GAACGTCTGG GAGCACATGG TATTCTGCTG ATGCCGCATT ATCTGACCGA AGCAAGCCAG GAAGGTCTGG TTGAACACGT TAAACAGGTG TGCAATGCCG TTAATTTTGG CGTGATCTTT TATAATCGTA GCGTGAGCAA ACTGAATGTT GATAGCCTGC AGCAGCTGGT TGAAAGCTGT CCGAATCTGA TTGGTTTTAA AGATAGCAGC GGTCAGATTG ATATGATGAC CGAAGTTGTT CAGACCCTGG GTGATCGTCT GAGCTATCTG GGTGGTCTGC CGACCGCAGA AATTTTTGCA GCACCGTATA AAGCACTGGG TTCTCCGGTT TATAGCAGCG CAGTGTTTAA CTTTATTCCG AAAACCGCCA TGGAATTTTA TAATGCCCTG CGCAATGATG ATTTTGCAAC CACCCAGCGT CTGATTCGTG ATTTTTTTCT GCCGCTGATT AAAATTCGTA ACCGTAAAAG CGGTTATGCA GTTAGCATGG TTAAAGCCGG TGCAAAAATT GTTGGTCATG ATGCCGGTCC GGTTCGTCCG CCTCTGAGCG ATCTGACACC GCAGGATTAT GAAGATCTGG CAGCACTGAT TGCAACCCTG GGTCCGCAG

**Figure S2** Codon optimized nucleotide sequence for the gene *kdg*D coding for the keto-deoxy glucarate dehydratases/decarboxylases (EC 4.2.1.41) from *C. testosteroni* KF-1:

ATGACACCGC AGGATCTGAA AGATGTTATG AGCAGCGGTC TGCTGAGCTT TCCGGTTACC GATTTTGATG CCCAGGGCAA TTTTAATGCA AAAGGTTATG CAGCACGTCT GGAATGGCTG GCACCGTATG GTGCAAGCGC ACTGTTTGCA GCCGGTGGCA CAGGTGAATA TTTTAGCCTG TATGGCGAAG AATATGGCCA GATTATCAAA ACCGCAGTTG ATACCTGTCG TGGTAAAGTT CCGATTATTG CCGGTGCCGG TGGTCCGACC CGTACCGCAA TTGCACATGC ACAGGAAGCA GAACGTCTGG GAGCACATGG TATTCTGCTG CTGCCGCATT ATCTGACCGA AGCCGGTCAG GAAGGTCTGA TTGCACACGT TGAACAGGTT TGCAAAAGCG TGAAATTTGG CGTGATTGTG TATAATCGTG ATCGTACCCG TTTTACACCG GAAAGCCTGG CAATTCTGGC AGAACGTTGT CCGAATCTGG TTGGTTTTAA AGATGGCATG GGCAATATTG AAACCATGAG CAGCATCTTT ATGAAAATGG GCGATCGTTT TGCATATCTG GGTGGTCTGC CGACCGCTGA AGTTTATGCA GCAGCATATA AAGCACTGGG TACACCGGTT TATAGCAGCG CAGTGTTTAA TTTTATTCCT CGCACCGCAA TGGCATTTTA TGAAGCCGTT CGTACCGATG ATATGGCAAC CCAGCATAAA CTGCTGAAAG AATTTTTTAT GCCGTATCTG AAAATTCGTA ATCGTGTGGA AGGTTATGGC GTTAGCATTA TTAAAGCCGG TGCAAAACTG GTTGGTCATG ATGCCGGTCC GGTTCGTGCA CCGCTGACCG ATCTGAAACC GGCAGAAATG GAAGAACTGA AAGTGCTGAT TGATAAACTG GGTCCGCAG

**Figure S3** Codon optimized nucleotide sequence for the gene *kdg*D coding for the keto-deoxy glucarate dehydratases/decarboxylases (EC 4.2.1.41) from *P. naphthalenivorans*:

ATGAATCCGC AGGATCTGAA AACCATTGTT AGCAGCGGTC TGCTGAGCTT TCCGGTTACC GATTTTGATG AACAGGGTGA TTTTCGTCCG AAAACCTATA TTGAACGTCT GGAATGGCTG GCACCGTATG GTGCAACCGC ACTGTTTGCA GCCGGTGGCA CAGGTGAATT TTTTAGCCTG ACCGGTGATG AATATCCGCT GATTATCAAA ACCGCAGTTA ATACCTGTGC CGGTAAAGTT CCGATTATTG CCGGTGTTGG TGGTCCGACC CGTTTTGCAA TTGCATGTGC ACAGGAAGCA GAACGTCTGG GAGCACATGG TATTCTGCTG CTGCCGCATT ATCTGATGGA AGCCGGTCAG GAAGGTCTGA TTGCACACGT TGAAGCAGTT TGCAAAAGCG TGAAATTTGG CGTGATTGTG TATAATCGCA ATGTTTGCAA ACTGACACCG GAAAGCCTGG CAATTCTGGC AGATCGTTGT CCGAATCTGA TTGGTTTTAA AGATGGCGTG GGCAATATTG AAACCATGAG CAGCATCTTT ATGAAAATGG GCGATCGTTT TAGCTATCTG GGTGGTCTGC CGACCGCTGA AGTTTATGCA GCAGCATATA AAGCACTGGG TACACCGGTT TATAGCAGCG CAGTGTTTAA CTTTATTCCG AAAACCGCCA TGGATTTTTA TCATGCCGTT GCATCTGATG ATCTGGCAAC CCAGCATCGT CTGCTGCGTG ATTTTTTTAT GCCGTATCTG GCCCTGCGTA ATAAAAATCC GGGTTATGCC GTTAGCATTG TTAAAGCCGG TGCAACCATT GTTGGTCATG ATGCCGGTCC GGTTCGTCCG CCTCTGACCG ATCTGAAACC GGCAGAAATG GAAGAACTGG CCGTTCTGAT TAAAAGCCTG GGTCCGCAG
